# Supplementary material for: Wavelength-tunable high-fidelity entangled photon sources enabled by dual Stark effects
Source: Nat Commun. 2024 Jul 10;15:5792. doi: 10.1038/s41467-024-50062-0 (PMC11237044; doi:10.1038/s41467-024-50062-0)
Supplement: Supplementary file 1 — Supplementary Information [file 41467_2024_50062_MOESM1_ESM.pdf]

Supplementary Information: Wavelength-tunable high-fidelity entangled photon sources enabled by dual Stark effects

Chen Chen, Jun-Yong Yan, Hans-Georg Babin, Jiefei Wang, Xingqi Xu, Xing Lin, Qianqian Yu, Wei Fang, Run-Ze Liu, Yong-Heng Huo, Han Cai, Wei E. I. Sha, Jiaxiang Zhang, Christian Heyn, Andreas D. Wieck, Arne Ludwig, Da-Wei Wang, Chao-Yuan Jin, and Feng Liu

CONTENTS

Supplementary Note 1. Sample structure 2

Supplementary Note 2. Deterministic preparation of the biexciton state 3

Supplementary Note 3. Linewidth measurement 3

Supplementary Note 4. Evaluation of two-photon entanglement fidelity 4

Supplementary Note 5. Time-resolved photoluminescence 5

Supplementary Note 6. Tuning of multiple quantum dots 6

Supplementary Note 7. Improvement in inhomogeneous broadening 7

Supplementary Note 8. Experimental setup 8

References 9

Supplementary Note 1. SAMPLE STRUCTURE

The heterostructure is grown by molecular beam epitaxy (MBE) technology on a [001]-oriented GaAs substrate. Quantum dots are embedded in an n-i-p diode structure, enabling effective manipulation of both the electric field experienced by the QDs and their charge states. The n-contact consists of Si-doped $Al_{0.15}Ga_{0.85}As$ with a doping concentration of 2×10^{18} cm^{-3}. A 20 nm $Al_{0.15}Ga_{0.85}As$ layer and a 10 nm $Al_{0.33}Ga_{0.67}As$ layer serve as a tunnel barrier to separate the QDs from the n-contact. GaAs quantum dots are grown in the $Al_{0.33}Ga_{0.67}As$ layer using the local droplet etching method, followed by a 273.6 nm $Al_{0.33}Ga_{0.67}As$ layer as the blocking barrier. The p-contact consists of 65 nm C-doped $Al_{0.15}Ga_{0.85}As$ (p+, doping concentration of 2×10^{18} cm^{-3}), 10 nm C-doped $Al_{0.15}Ga_{0.85}As$ (p++, doping concentration of 8×10^{18} cm^{-3}), and 5 nm C-doped GaAs (p++, doping concentration of 8×10^{18} cm^{-3}). Below the n-i-p diode structure, a distributed Bragg reflector consisting of 10 pairs of AlAs (67.08 nm thick)/$Al_{0.33}Ga_{0.67}As$ (59.54 nm thick) is grown to enhance the collection efficiency of photons. The overall structure is illustrated in Supplementary Fig. 1.

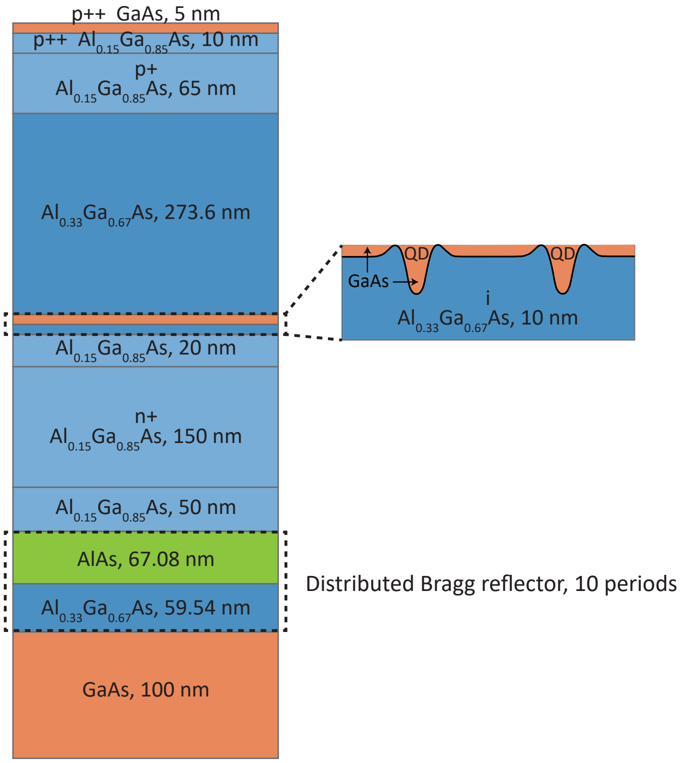

Supplementary Figure 1. **Schematic diagram of sample structure.**

Supplementary Note 2. DETERMINISTIC PREPARATION OF THE BIEXCITON STATE

We measure the power dependence of the integrated intensity of XX and X (see Supplementary Fig. 2), showing well-defined Rabi oscillations. Setting the pump power such that the inversion of the quantum dot from $|G\rangle$ to $|XX\rangle$ is most probable (π pulse), and we can deterministically prepare $|XX\rangle$ [1, 2].

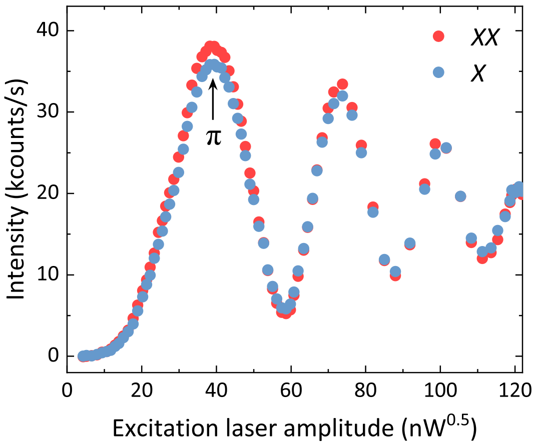

Supplementary Figure 2. **The integrated intensity of XX (red) and X (blue) as a function of the square root of the TPE pump power.**

Supplementary Note 3. LINEWIDTH MEASUREMENT

To measure the linewidth of the neutral exciton (X) emission, we perform resonance fluorescence measurements by sweeping the bias (V_{g}) at a fixed wavelength of a narrow-bandwidth CW laser (100 kHz linewidth) [3]. Changing the bias causes the Stark shift of X across the fixed CW laser wavelength and converts the corresponding bias into energy (shown at the top as an additional x-axis in Supplementary Fig. 3). Fitting with the bimodal Lorentzian function, the linewidths of QD A and QD B are 5.37 μeV and 4.24 μeV, respectively.

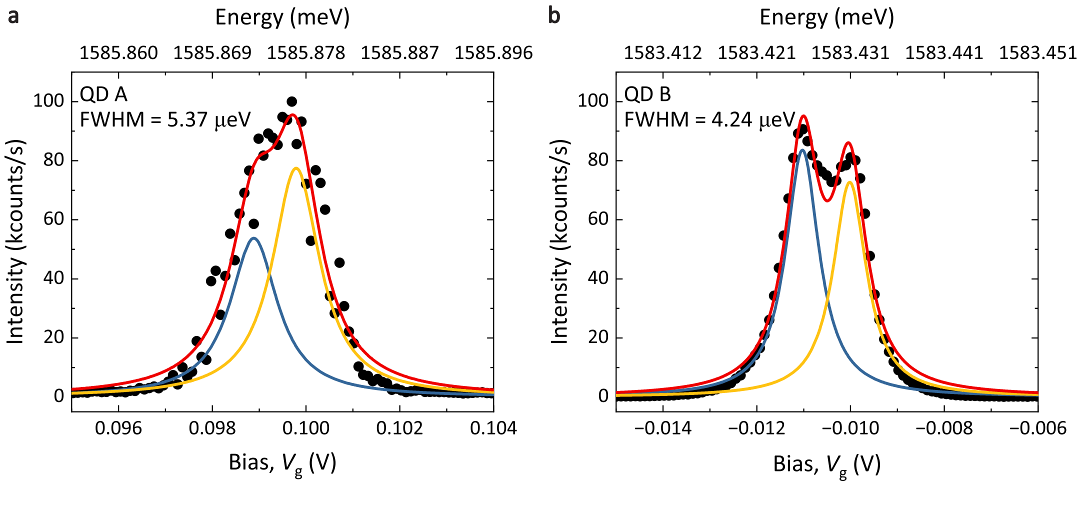

Supplementary Figure 3. **Resonance fluorescence spectra of the neutral exciton emission.** **a**, Resonance fluorescence from QD A and **b**, QD B. The linewidths of QD A and QD B are 5.37 μeV and 4.24 μeV, respectively, obtained by fitting with bimodal Lorentzian function (red line). The measurement is performed by sweeping the bias at a fixed CW laser wavelength. The blue and yellow lines in (**a**) and (**b**) represent the two peaks of bimodal fit.

Supplementary Note 4. EVALUATION OF TWO-PHOTON ENTANGLEMENT FIDELITY

When performing cross-correlation measurements using a reduced set of projective measurements [4], we calculate the entanglement fidelity of the measured quantum state with respect to $\frac{1}{\sqrt{2}}(|H_{\mathrm{XX}}H_{\mathrm{X}}\rangle + |V_{\mathrm{XX}}V_{\mathrm{X}}\rangle)$ according to:

$$f = (1 + C_{\mathrm{linear}} + C_{\mathrm{diagonal}} - C_{\mathrm{circular}})/4, \tag{1}$$

where C_{linear}, C_{diagonal}, and C_{circular} are the degrees of correlation measured on linear, diagonal, and circular bases, respectively. The degree of correlation for a given basis (μ) is defined as:

$$C_\mu = \frac{g^{(2)}_{\mathrm{XX,X}} - g^{(2)}_{\mathrm{XX,\overline{X}}}}{g^{(2)}_{\mathrm{XX,X}} + g^{(2)}_{\mathrm{XX,\overline{X}}}}, \tag{2}$$

$g^{(2)}_{\mathrm{XX,X}}$ and $g^{(2)}_{\mathrm{XX,\overline{X}}}$ are cross-correlation measurements for co-polarized and cross-polarized bases, respectively. Supplementary Table 1 demonstrates the correlations under different conditions of QD A mentioned in the main text.

		QD A						
	V_g (V) / C_μ	0.02	0.06	0.1	0.14	0.18	0.2	0.24
Without FSS tuning	C_{linear}	0.946(2)	0.970(2)	0.978(1)	0.978(1)	0.971(1)	0.962(2)	0.961(2)
	C_{diagonal}	0.483(16)	0.385(19)	0.414(29)	0.373(21)	0.354(28)	0.414(21)	0.636(16)
	C_{circular}	-0.503(14)	-0.369(29)	-0.350(19)	-0.328(19)	-0.362(24)	-0.415(22)	-0.618(22)
With FSS tuning	C_{linear}	0.949(2)	0.949(2)	0.949(3)	0.952(2)	0.961(2)	0.952(3)	0.964(2)
	C_{diagonal}	0.941(3)	0.952(3)	0.939(3)	0.935(3)	0.900(4)	0.919(3)	0.965(2)
	C_{circular}	-0.946(2)	-0.918(4)	-0.941(2)	-0.943(2)	-0.962(2)	-0.961(2)	-0.949(2)

Supplementary Table 1. **Correlations for the different polarization basis with and without FSS tuning.**

Supplementary Note 5. TIME-RESOLVED PHOTOLUMINESCENCE

We measure the photoluminescence (PL) decay of XX emission under two-photon excitation and X emission under phonon-assisted excitation using a single-photon avalanche diode (IRF $\sim$ 68 ps with a weak tail). Fitting with a single exponential function, the lifetimes of XX and X at 0.1 V are 181(3) ps and 255(11) ps, respectively. The lifetimes of XX and X under different biases are shown in Supplementary Fig. 4b.

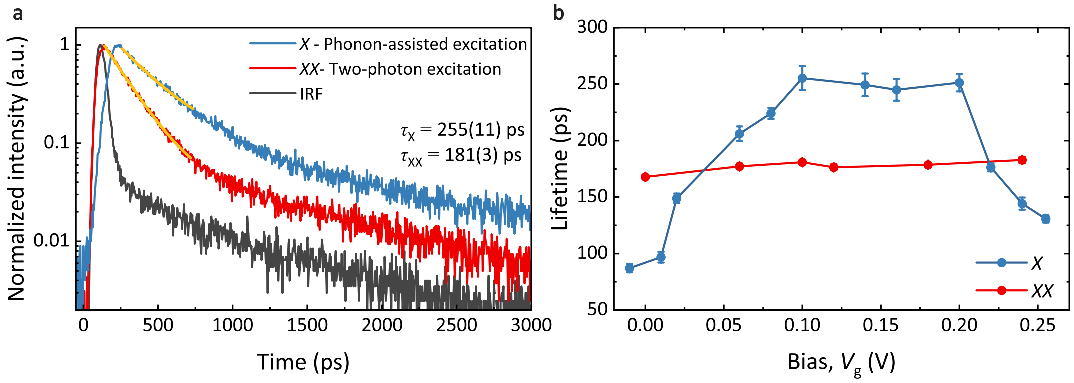

Supplementary Figure 4. **Lifetime measurement of QD A. a**, Normalized time-resolved PL of biexciton (XX, red) emission measured under two-photon excitation and exciton (X, blue) emission measured under phonon-assisted excitation at V_{g} = 0.10 V. The lifetime of XX and X extracted by single exponential fitting (yellow) are τ_{XX} = 181(3) ps and τ_{X} = 255(11) ps, respectively. Black: instrument response function (IRF). **b**, The lifetimes of XX and X as a function of bias. Error bars show the standard error for single exponential fitting residuals.

Supplementary Note 6. TUNING OF MULTIPLE QUANTUM DOTS

To demonstrate the feasibility of our scheme in achieving wavelength matching for multiple high-quality quantum dots, we characterize two QDs (QD B and QD C) whose wavelength tuning ranges for the X state overlap with that of QD A as mentioned in the main text. Supplementary Figs. 5a-c show the bias-dependent photoluminescence spectra of the X states of the three QDs. By carefully scanning the bias, we tuning the X emission wavelengths of QD B and QD C to match that of QD A (indicated by the red dashed line). At the resonance wavelength, the FSS of three QDs can be tuned to close to zero by applying appropriate CW laser power, as shown in Supplementary Figs. 5d-f.

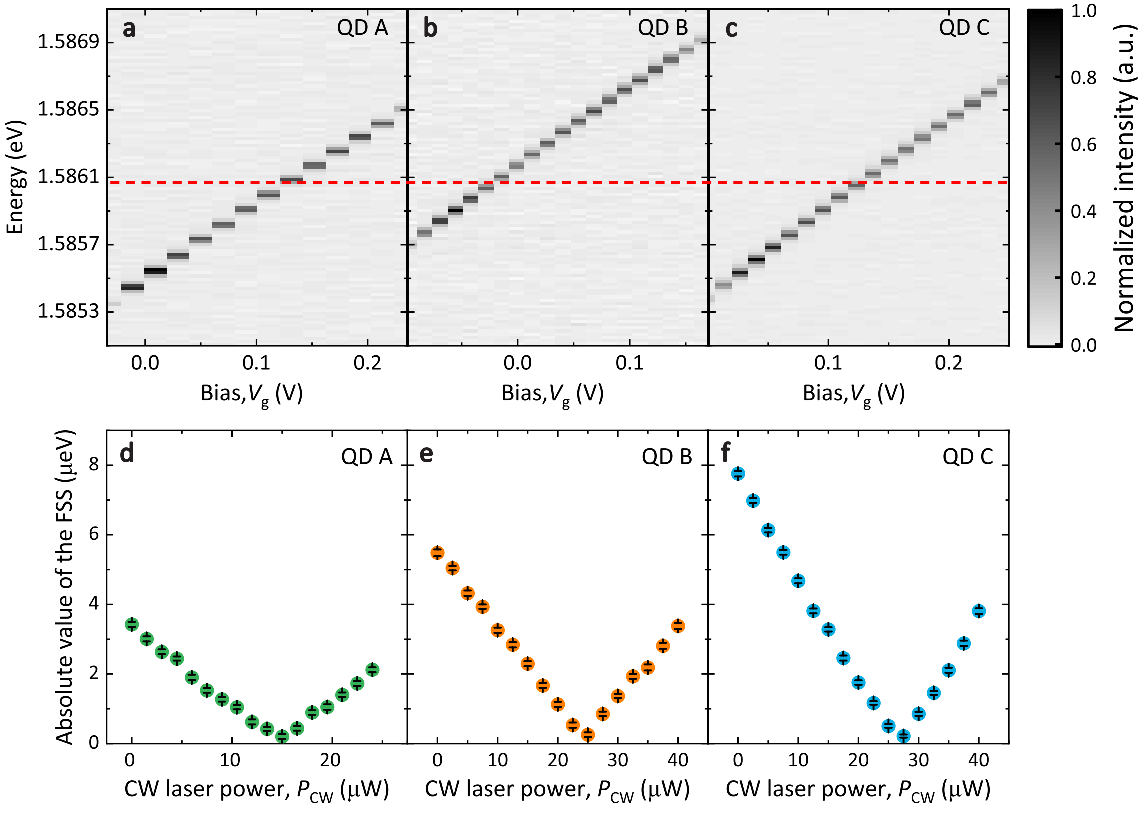

Supplementary Figure 5. **Tuning of multiple QDs. a-c**, Bias-dependent photoluminescence spectra of the X state for QD A, QD B, and QD C. Red line: indicate the wavelength of three QDs at resonance. **d-f**, FSS of QD A, QD B, and QD C as a function of CW laser power. Error bars reflect the standard error for sine fitting residuals.

Supplementary Note 7. IMPROVEMENT IN INHOMOGENEOUS BROADENING

To reduce the intrinsic non-uniformity in QD emission wavelengths, we perform deeper etching and optimize growth parameters. The wavelength distribution of 794 randomly selected QDs on the new sample is illustrated in Supplementary Fig. 6, with a 3.97 meV inhomogeneous broadening linewidth. These results significantly increase the number of QDs that can be tuned to resonance.

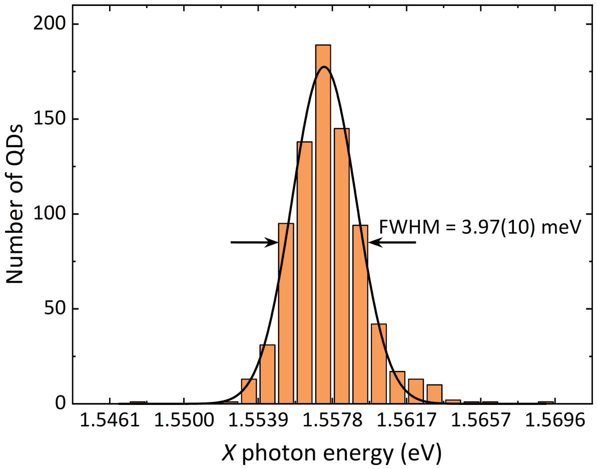

Supplementary Figure 6. X **photon energy distribution of 794 randomly selected QDs on a deeper etched sample.** Black line: fitting with a Gaussian function.

Supplementary Note 8. EXPERIMENTAL SETUP

The schematic of our experimental setup is shown in Supplementary Fig. 7. The QD sample is placed in a 3.6 K closed-loop cryostat. Using a confocal microscope, the TPE laser and the CW-tuned laser are applied to the sample through one arm, and the emitted entangled photon pairs are collected into a single-mode fiber through the other arm. The Ti: Sapphire laser (80 MHz, 140 ps) is shaped into a pulse with a duration of $\sim$ 6 ps by a homemade pulse shaper [5] for TPE. The collected TPE laser and CW laser scattered from the sample surface are primarily filtered by four tunable notch filters. Any remaining laser background is further removed by a filter based on volume phase holographic (VPH) transmission grating. The X and XX photons are dispersed by the same VPH transmission grating. Two quarter-wave plates (QWPs) and a half-wave plate (HWP) are used to compensate the polarization rotation introduced by the optical components [6]. Before X and XX are coupled into the single-mode fiber, a QWP, a HWP and a polarizer are inserted into each beam to set different projection states.

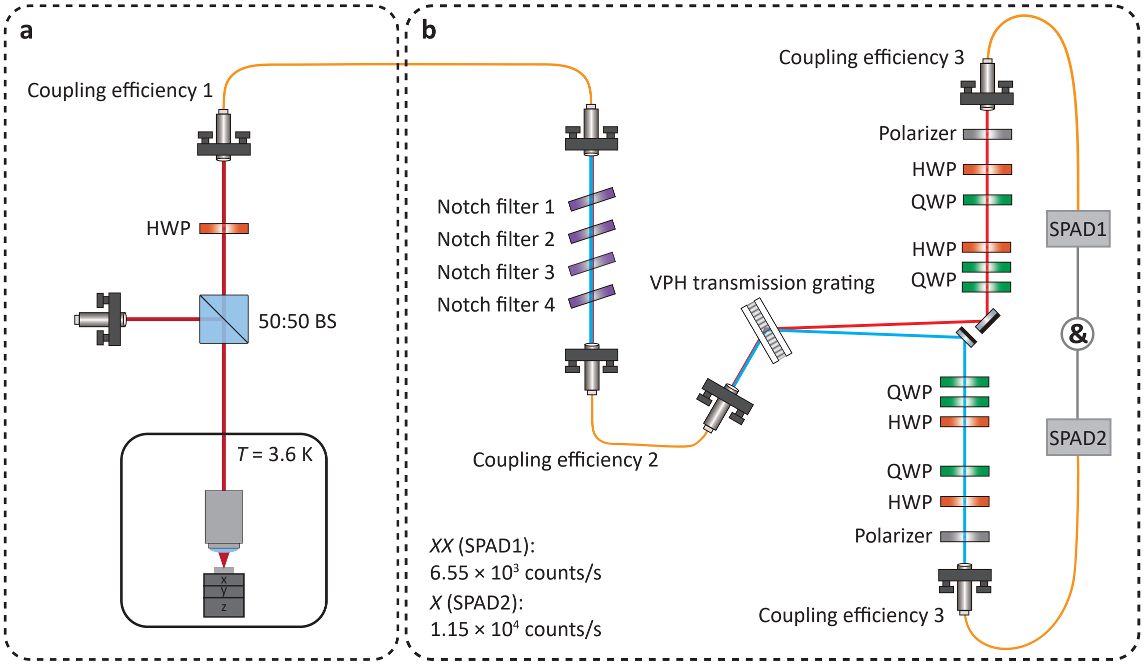

Supplementary Figure 7. **Schematic of the experimental setup. a**, A confocal microscope setup to excite the QD and collect the emitted photons. **b**, Filtering and entanglement analysis. The TPE laser and the CW-tuned laser are filtered by four tunable notch filters and further removed by a grating-based filter. The X and XX photons are dispersed by a VPH transmission grating and coupled into single-mode fiber respectively. The HWP, QWP and polarizer placed in front of the fiber are used to set the projection state.

At π pulse excitation condition, the count rates of X and XX we finally detected on the single-photon avalanche diode (SPAD) are 1.15×10^4 counts/s and 6.55×10^3 counts/s, respectively. The reason for the lower count rate of XX is that the wavelength of the CW laser that tunes the FSS is very close to the wavelength of XX, and XX will be slightly filtered out when the notch filter (bandwidth $\sim$ 200 pm) is used to remove the CW laser. Supplementary Table 2 demonstrates the transmission efficiency of the optical elements in the setup.

Optical path	Transmission	
	X	XX
Objective	0.889	
Optical Window	0.926	
50:50 BS	0.498	
HWP 1	0.984	
Coupling efficiency 1	0.550	
Notch filter 1	0.925	0.844
Notch filter 2	0.981	0.980
Notch filter 3	0.980	0.824
Notch filter 4	0.984	0.882
Coupling efficiency 2	0.782	0.709
Grating filter	0.774	0.763
Coupling efficiency 3	0.544	0.465
**Setup efficiency**	**0.064**	**0.034**

Supplementary Table 2. **Transmission of the optical elements in the setup.**

[1] Jayakumar, H. *et al.* Deterministic Photon Pairs and Coherent Optical Control of a Single Quantum Dot. *Physical Review Letters* **110**, 135505 (2013). URL `https://link.aps.org/doi/10.1103/PhysRevLett.110.135505`. 1211.2613.

[2] Müller, M., Bounouar, S., Jöns, K. D., Glässl, M. & Michler, P. On-demand generation of indistinguishable polarization-entangled photon pairs. *Nature Photonics* **8**, 224–228 (2014). URL `https://www.nature.com/articles/nphoton.2013.377`. 1308.4257.

[3] Stufler, S., Ester, P., Zrenner, A. & Bichler, M. Power broadening of the exciton linewidth in a single InGaAs/ GaAs quantum dot. *Applied Physics Letters* **85**, 4202–4204 (2004). URL `https://pubs.aip.org/apl/article/85/18/4202/939989/Power-broadening-of-the-exciton-linewidth-in-a`.

[4] Hudson, A. J. *et al.* Coherence of an Entangled Exciton-Photon State. *Physical Review Letters* **99**, 266802 (2007). URL `https://link.aps.org/doi/10.1103/PhysRevLett.99.266802`. 0707.3556.

[5] Yan, J. *et al.* Double-Pulse Generation of Indistinguishable Single Photons with Optically Controlled Polarization. *Nano Letters* **22**, 1483–1490 (2022). URL `https://pubs.acs.org/doi/10.1021/acs.nanolett.1c03543`. 2109.09279.

[6] Zhou, L. *et al.* Polarization Calibration Scheme for a Practical Handheld Free Space Quantum Key Distribution Link. In *2019 IEEE Globecom Workshops (GC Wkshps)*, 1–5 (IEEE, 2019). URL `https://ieeexplore.ieee.org/document/9024509/`.
